# DPY30 Promotes Proliferation and Cell Cycle Progression of Colorectal Cancer Cells via Mediating H3K4 Trimethylation

**DOI:** 10.7150/ijms.80073

**Published:** 2023-05-11

**Authors:** Wei-Chao Su, Xiao-Mei Mao, Si-Yang Li, Chun-Ying Luo, Rui Fan, Hai-Feng Jiang, Lin-Jun Zhang, Ya-Tao Wang, Guo-Qiang Su, Dong-Yan Shen

**Affiliations:** 1 Department of Colorectal Tumor Surgery, The First Affiliated Hospital of Xiamen University, School of Medicine, Xiamen University, Xiamen 361003, Fujian Province, P.R. China.; 2 School of Pharmaceutical Science and Technology, Suzhou Chien-Shiung Institute of Technology, Suzhou 215411, Jiangsu Province, P.R. China.; 3 Xiamen Cell Therapy Research Center, The First Affiliated Hospital of Xiamen University, School of Medicine, Xiamen University, Xiamen 361003, Fujian Province, P.R. China.; 4 Department of Pathology, Affiliated Hospital of Youjiang Medical University for Nationalities, Baise 533000, Guangxi Province, P.R. China.; 5 Medical College, Guangxi University, Nanning 530004, Guangxi Province, P.R. China.

**Keywords:** DPY30, Colorectal carcinoma, Cell proliferation, Cell cycle, H3K4 trimethylation

## Abstract

DPY30, a core subunit of the SET1/MLL histone H3K4 methyltransferase complexes, plays an important role in diverse biological functions through the epigenetic regulation of gene transcription, especially in cancer development. However, its involvement in human colorectal carcinoma (CRC) has not been elucidated yet. Here we demonstrated that DPY30 was overexpressed in CRC tissues, and significantly associated with pathological grading, tumor size, TNM stage, and tumor location. Furthermore, DPY30 knockdown remarkably suppressed the CRC cell proliferation through downregulation of PCNA and Ki67 *in vitro* and *in vivo*, simultaneously induced cell cycle arrest at S phase by downregulating Cyclin A2. In the mechanistic study, RNA-Seq analysis revealed that enriched gene ontology of cell proliferation and cell growth was significantly affected. And ChIP result indicated that DPY30 knockdown inhibited H3 lysine 4 trimethylation (H3K4me3) and attenuated interactions between H3K4me3 with PCNA, Ki67 and cyclin A2 respectively, which led to the decrease of H3K4me3 establishment on their promoter regions. Taken together, our results demonstrate overexpression of DPY30 promotes CRC cell proliferation and cell cycle progression by facilitating the transcription of PCNA, Ki67 and cyclin A2 via mediating H3K4me3. It suggests that DPY30 may serve as a potential therapeutic molecular target for CRC.

## Introduction

Colorectal carcinoma (CRC) is one of the most common gastrointestinal malignancies, currently ranks third in the incidence of malignant tumors in the world, and is one of the leading causes of cancer-related death 1. Surgery, chemotherapy and radiation therapy are routine treatments for CRC 2. These treatments can be used in combination, depending on the location and progression of the tumor. Chemotherapy has many side effects because it kills any cell that is proliferating or dividing. In addition, there are many cases of relapse each year 3. Although some new findings have been obtained in the pathogenesis of CRC recently, its molecular mechanism still needs to be further elucidated clearly and systematically. Diagnostic markers are used for risk stratification and early detection, which might prolong overall survival. Nowadays, the widespread use of semi-invasive endoscopic methods and fecal blood tests characterized by suboptimal accuracy of diagnostic results has led to the detection of cases at later stages. The identification of “ideal” diagnostic biomarkers, having high sensitivity and specificity, being safe, cheap and easy to measure, remains a challenge. More importantly, accurate and effective cancer treatment methods or strategies for CRC can be developed based on these markers. The development of new diagnostic tumor markers and accurate and effective cancer treatment methods or strategies need to be studied urgently.

Inherent genetic alterations are key drivers of cancer through dynamic epigenetic regulation of gene expression. Chromatin modifications, including histone methylation, acetylation, phosphorylation, ubiquitination, and so on, have been reported to play a key role in regulating gene expression 4. For histone methylation, histone H3 lysine 27 trimethylation (H3K27me3) function is associated with gene repression 5, while SET1/MLL complex-mediated H3K4 methylation is associated with gene activation 6. Histone methylation modification includes H3K4me1, H3K4me2 and H3K4me3, which have different biological functions. H3K4me3 definitively marks active and poised transcription start sites, H3K4me2 marks active gene body, and H3K4me1 marks active and poised enhancers 7, 8. With the deepening of research, more and more studies have shown that H3K4me1/2/3 has many differences in both distribution patterns and functions 9. In mammals, methylation of H3K4 is catalyzed by SET1/MLL histone methyltransferase complex (SET1/MLL complex, or KMT) and is related to gene activation. SET1/MLL complexes are composed of catalytic subunit (SET1A/SET1B/MLL1-4) and core subunits including WDR5, RBBP5, ASH2L, and DPY30. All core subunits together also called as WRAD, which is an evolutionarily conserved subcomplex, are required for SET1A methyltransferase activity 10. According to the report 11, WDR5 and RBBP5 are vital for all three kinds of methylations of H3K4 (H3K4me1, H3K4me2, and H3K4me3), while ASH2L and DPY30 are primarily necessary for H3K4me3.

DPY30 plays a key role in regulating H3K4 methylation and also has important physiological functions in development and disease 12. It was shown that as a core subunit, DPY30 directly interacted with ASH2L. DPY30 was first identified in *C. elegans* 13, then previous studies on DPY30 had focused on stem cell function, cell senescence, adhesion, migration and invasion 14, 15. Gradually, its role in the development of tumors has been explored. In cholangiocarcinoma 16, DPY30 was one of the causes of tumorigenesis and poor prognosis of patients. Moreover, reports also demonstrated that DPY30 was related to the proliferation, migration, and invasion of gastric cancer cells 17. Bing Liu showed that the mRNA expression level of DPY30 was significantly increased in hepatocellular carcinoma tissues 18. Recently, it has been demonstrated that ABHD5 inhibited c-Met activation to reduce CRC stemness via interacting with DPY30 thereby inhibiting its nuclear translocation and activity of SET1A 19. Although advances in research knowledge related to DPY30 have been increased recently, the expression profile and biological function of DPY30 in CRC are still largely unknown and remain to be further elucidated.

Here, we interrogated the functional role of DPY30 in colorectal cancer cells proliferation *in vitro* and *in vivo*. Overexpression of DPY30 played an important role in promoting CRC cell proliferation and cell cycle progression, which was associated with its establishment of H3K4me3 level and epigenetic modification on PCNA, Ki67 and cyclin A2 promoters. Therefore, DPY30 could be a promising target in the treatment of human colorectal carcinoma.

## Materials & Methods

### Chemicals and antibodies

The antibody against DPY30 used for IHC was from Abcam (ab126352). The primary antibodies used for WB and IHC included: PCNA (Santa Cruz, sc-25280), Ki67 (Santa Cruz, sc-23900), cyclin A (Santa Cruz, sc-271682). And other antibodies applied to WB as follows: α-actinin (Proteintech, 11313-2-AP), β-tubulin (Signalway Antibody, #48885), H3K4me3 (CST, C42D8, #9751), Histone H3 (CST, D1H2, **#**4499), cyclin B1(sc-245), cyclin D1 (sc-8396), cyclin E (sc-377100), p21 (sc-6246). Anti-mouse or rabbit secondary antibodies were purchased from Sigma. FBS, high glucose DMEM medium, and Trypsin (with EDTA) were purchased from Gibco (Thermo Fisher Scientific, USA). penicillin-streptomycin purchased from BasalMedia. Other reagents were all analytical grade. Other relevant kits or special supplies will be described below.

### Cell culture and construction of stable knockdown cell lines

The wild-type human CRC cell lines LOVO, RKO, SW620, HCT116, HT29, SW480, caco2, KM12C, normal colonic epithelial cell NCM460 were donated from the relevant laboratories of the School of Life Sciences and School of Medicine, Xiamen University, then were cultured and frozen in liquid nitrogen in the Xiamen Cell Therapy Research Center, The First Affiliated Hospital of Xiamen University. Cells were cultured in DMEM medium supplemented with 10 % FBS, 100 U/mL penicillin and 100 μg/mL streptomycin in cell incubator (37°C 5% CO_2_). The shRNA for DPY30 plasmid and the control vector PLKO were bought from Public Protein/Plasmid Library (Geneppl, co, Ltd.). The generation of retrovirus supernatants, transfection for stable DPY30 knockdown cell lines HT29, SW480, caco2 and KM12C, then selected by puromycin for 7 days were prepared as described previously 16.

### Patients and tumor specimens

CRC tissue samples, para-cancerous tissues, and distal normal tissues were obtained and immediately cryopreserved in liquid nitrogen after surgical resection from CRC patients. They all under curative resection with informed consent and write informed consent at the Department of Colorectal Tumor Surgery, First Affiliated Hospital of Xiamen University. The tissue microarray of 94 CRC tissues sections and 86 paired paratumor tissues sections purchased from Shanghai Outdo Biotech Company (Cat No. HColA180Su19). Our study was approved by the Ethics Committees of The First Affiliated Hospital of Xiamen University (The IRB approval number: XMYY-2022KYSB097) Xiamen, China.

### Western blotting

Cells or tissues samples were fully lysed in RIPA buffer containing protease inhibitor cocktail (Roche), phosphatase inhibitor cocktail (Roche) and PMSF (Solarbio). Then centrifugated 15 min, 12000 rpm, 4°C. The concentration of protein was detected by BCA assay (Pierce). The lysate for each sample (10 μg) was separated by SDS-PAGE, then transferred to PVDF membranes (Millipore) using eBlot protein transfer system (GenScript). Then block with 5% skim milk, PVDF membranes were incubated with indicated antibody 4°C overnight. Then incubated and conjugated with secondary antibody for 1h at room temperature, followed by detection using enhanced chemiluminescent regent (Biothrive). and imaged with a Bio-Rad imaging system.

### Immunohistochemistry (IHC)

The formalin-fixed paraffin-embedded tissues were dewaxed then repaired by high pressure with the pressure cooker by boiling in citrate buffer, incubating in 3% H_2_O_2_ peroxidase blocker buffer and 10% donkey serum to block. Then the slides were incubated with antibody overnight at 4°C. DAB Kit (Maxim Biotechnologies) were then employed to visualize the staining proteins. A relative score was assigned to the staining results according to the intensity of IHC staining and the proportion of tumor cells with positive response. The staining intensity combine with number of positive cells were used to calculate the IHC score, values 0, 1, 2 and 3. The staining index (value, 0-12) was determined from the score obtained by staining intensity and positive area. The correlation between DPY30 and Ki67, PCNA, cyclin A were measured with the IHC score values and undergo to Pearson correlation analysis.

### Quantitative RT-PCR

Total RNA was extracted from cells or tissue using RNAsimple Total RNA kit (TIANGEN, DP419, China) on the basis of the instructions, and RNA were reverse transcribed into cDNA with FastQuant RT kit (with gDNase) (TIANGEN, KR106). Then relative mRNA expression levels were analyzed by qRT-PCR using SuperReal PreMix Plus kit (TIANGEN, FP205) with ABI 7500 fast fluorescence temperature cycler. The primers designed showed in Table 1. The relative mRNA expression levels of genes were calculated using the 2^-△△Ct^ method compared to internal reference. Each experiment repeated three times.

### Cell proliferation assay

Changes in cell proliferation were performed using CCK-8 (Cell Counting Kit-8) kit (MedChemExpress, China). 5 × 10^3^ CRC cells per well were seeded in 96-well microplates overnight. After 24 h, 48 h, 72 h or 96 h, respectively, CCK-8 solution was added in each well. After incubation for 3 hours, the OD absorbance was measured at 450 nm and normalized mean OD were calculated.

### Cell cycle analyses

The CRC cells were treated starved for 0 h, 12 h and 24 h to synchronize the cell cycle respectively, and grew in complete medium for 24 h. The method of serum-free starvation was used. Next, the cells were harvested, fixed and stained with propidium iodide gradually. See reference 16 for specific staining methods. Three parallel experiments were performed.

### RNA isolation and sequencing and transcriptome analysis

Three independent replicates of HT29 cells with stable DPY30 knockdown were plated and cultured in 10-cm dish for 24 h, as well as HT29-shCtrl cells. Then washed in cold PBS and total RNA was extracted using Trizol reagent kit. The RNA sequencing and data analysis were commissioned and performed by Gene Denovo Biotechnology Co. (Guangzhou, China). The integrity and quality of the RNA were assessed and then enriched. The enriched mRNA was fragmented and reversely transcribed into cDNA. After end repaired and purified, cDNA library was sequenced using Illumina Novaseq6000 by Gene Denovo Biotechnology Co.

Differentially expressed genes (DEGs) were performed by DESeq2 software between six samples and performing the comparison between shCtrl and shDPY30. False discovery rate (FDR, adjusted *p*-value) ≤ 0.05 and fold change ≥ 2 were considered to filtrate genes. Then we screened the top 50 downregulated and upregulated genes, following heatmaps were generated and designed via Omicsmart online platform. GO enrichment analysis was performed in the Gene Ontology database, gene numbers were counted for each GO term, significantly enriched GO terms in DEGs comparing to the genome background, which revealed the primary biological functions of DEGs. Moreover, the correlation analysis was performed by R. Correlation of two parallel experiments provides the evaluation of the reliability of experimental results as well as operational stability to evaluate repeatability between samples. They all to achieve the claimed statistical power.

### Chromatin immunoprecipitation (ChIP) assay

ChIP assay was performed using the SimpleChIP Plus Sonication Chromatin IP Kit (Cell Signaling Technology, USA). HT29 cells were cross-linked with formaldehyde, then lysed and sonicated. Protein samples were incubated at 4°C with anti-H3K4me3 antibody or anti-rabbit IgG. The complexes were precipitated with Protein G Magnetic Beads for 2 h. After washes, chromatin from Antibody/Protein G Magnetic Beads was eluted and reversed cross-links. Then DNA was purified and amplified by qPCR with primer pairs (Table 1). Antibodies were also purchased from Cell Signaling Technology and used at recommended concentrations.

### *In vivo* animal model

6-8-week-old male nude mice (BALB/c, 18-20 g) were obtained from the Xiamen University Laboratory Animal Center. All mice were raised in specific-pathogen-free conditions under 12/12 cycle of light at room temperature (25-27°C) and allowed libitum access to food and water in the animal room of the laboratory in The First Affiliated Hospital of Xiamen University. Mice were randomly divided into two groups (n = 6 per group, total number of animals: 12): shCtrl group and shDPY30 group. 5×10^6^ HT29 cells (shCtrl or shDPY30) were suspended with PBS and injected subcutaneously into the dorsal of each mouse respectively. Tumor volumes were measured every 2 days and calculated with the formula V (mm^3^) = 1/2 (length × width^2^). After 21 days, mice were sacrificed by cervical dislocation after anesthetized with 2% isoflurane, then the xenografted tumors were removed for further analysis including tumors photographed, tumors weight measured, IHC staining, protein expression of PCNA, Ki67 and cyclin A. For AOM/DSS mouse model, established according to the report 20. We used 6-8 weeks old mice for induction of CRC (n = 24). Mice were injected 12.5 mg/kg AOM intraperitoneally. Then mice recovered for 5 days and were administered 2.5 % DSS in drinking water for 5 days. Let the mice recovered for 2 weeks. Repeat DSS induction twice. Mice were anesthetized with isoflurane and sacrificed by cervical dislocation at 72^th^ day after the final DSS cycle. IHC staining to evaluate the expressions of DPY30. The left ears of all nude mice were marked with stud number and we record the group allocation. Animal experiments were performed according to ethical guidelines of animal experiment and reviewed and approved by the Institutional Animal Care and Use Ethics Committee of Xiamen University.

### Statistical analysis

Data were represented as the mean ± SEM from at least three independent experiments and statistical analyses with GraphPad Prism version 8.0.1. Two-tailed Student's t-tests and ANOVA were used to analyze the data. The correlation between DPY30 expression and clinicopathologic parameters was assessed using the Chi-square (χ2) test. The correlation between IHC scores of DPY30 and other protein level was evaluated by Pearson correlation analysis. The difference was considered to be statistically significant at *P < 0.05, **P < 0.01, ***P < 0.001.

## Results

### Clinical significance and overexpression of DPY30 in CRC tissues

**Figure 1:**
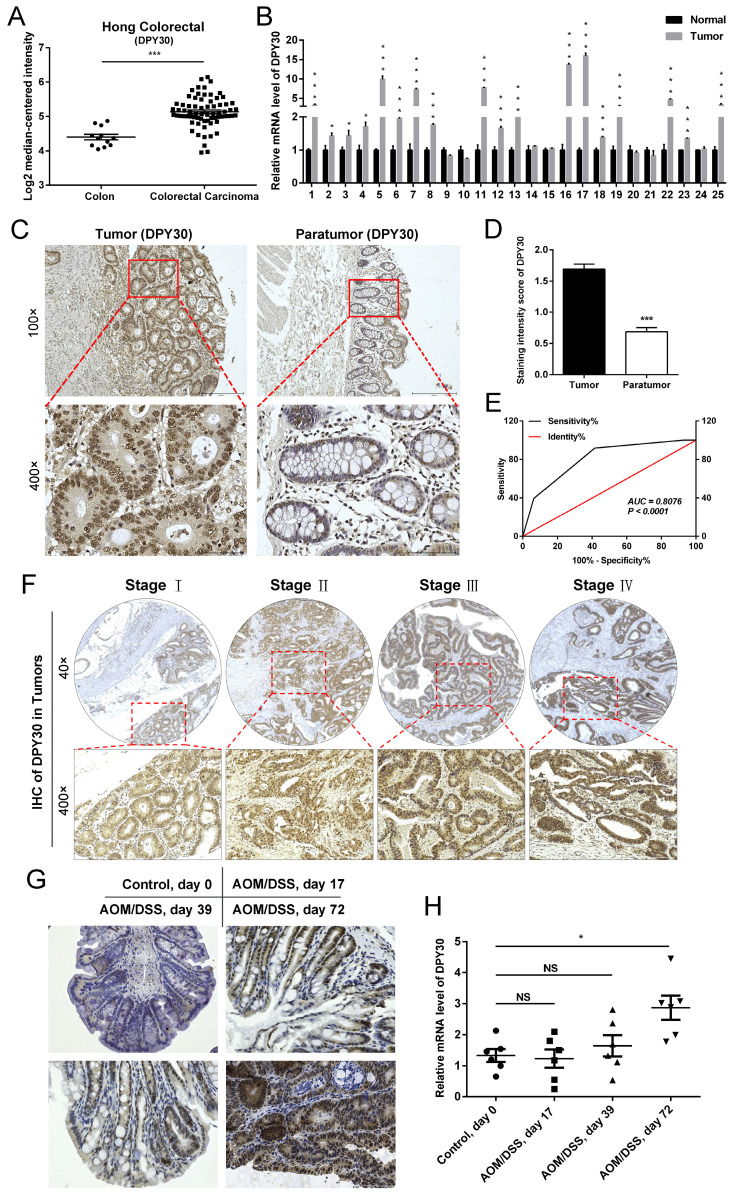
** DPY30 is upregulated in human colorectal carcinoma (CRC) and AOM/DSS mouse model of inflammatory CRC.** (A) The mRNA expressions level of DPY30 in normal colon tissues (n = 12) and CRC tissues (n = 70) from Hong Colorectal dataset of the Oncomine database. (B) qRT-PCR to detect the mRNA expressions level of DPY30 in clinical CRC tumor and paired normal tissues samples (n = 25). (C) Representative IHC images of DPY30 in a tissue microarray were shown (Tumor, n = 94. Paratumor, n = 86). Magnification, 100×, 400×. (D) IHC intensity score of DPY30 in tumor and paratumor microarray. (E) Receiver operating characteristic (ROC) analysis was used to examine the diagnostic value and accuracy of DPY30 for CRC. (F) Representative IHC staining of DPY30 in different CRC TNM grades (AJCC Ⅰ, Ⅱ, Ⅲ and Ⅳ). Magnification, 40×, 200×. (G) IHC staining of DPY30 was performed in the colon of AOM/DSS treated mice on day 0, 17, 39 and 72. Magnification, 200×. (H) The mRNA expressions level of DPY30 were evaluated in different stages of AOM/DSS model. Data were expressed as mean ± SEM. NS, no significant difference, *P < 0.05, ***P < 0.001.

According to the Oncomine database (*https://www.oncomine.com/*), the expression of DPY30 was appreciably upregulated in CRC tissues (n=70) compared with that in normal colon tissues (n=12) (Fig.1A). Consistent with these biostatistics, it was found that the mRNA expression level of DPY30 in clinical CRC tissue samples was higher than those in adjacent normal tissues significantly by qPCR. Of the 25 CRC samples, 72% of tumor samples presented DPY30 overexpressed (Fig. 1B). Tissue microarrays were also used to analyze DPY30 expression on 94 CRC tissues and 86 paired paratumor tissues by IHC staining, and demonstrated that DPY30 expression was much higher in the tumor tissue than in paratumor tissue, and mainly localized in the cell nucleus. (Fig. 1C).

In order to assess the level of DPY30 in these two kinds of tissues, the staining intensity of DPY30 showed in Table 2. The high expression rate of DPY30 in tumor tissues was 58.51% (55/94 cases), which was much higher than in paratumor tissues (8.14%, 7/86 cases). Consistently, staining intensities in tumor tissues showed higher scores than in paratumor tissues (Fig. 1D). According to the scores, receiver operating characteristic (ROC) analysis was applied to examine the diagnostic value and accuracy of DPY30 in classifying patients, which showed the areas under the curves (AUC) was 0.8076 *(P* < 0.0001) (Fig. 1E). Upon clinicopathological characteristics correlation analysis, elevated DPY30 protein levels positively correlated with pathological grading (*P* = 0.0348), tumor size (*P* = 0.0192), TNM stage (*P* = 0.0365), and tumor location (*P* = 0.019) in CRC patients (Table 3). As for the correlation of DPY30 with the actual tumor grade/stage, the IHC staining of DPY30 in different CRC TNM grades (AJCC Ⅰ, Ⅱ, Ⅲ and Ⅳ) (Fig. 1F) revealed that the expression level of DPY30 was positively correlated with TNM grades.

The AOM/DSS mouse model of inflammatory CRC was successfully established according to the previous study 21. As shown in Fig. 1G, colon tissues undergoing the process displayed different pathological features from the morphology observation at the time of drug induction. On the 72^nd^ day colon tumor formed, DPY30 exhibited deep staining with the disordered organizational structure, the cell morphology and arrangement compared to the control model of noninflammatory colon mice without AOM/DSS induction (Control, Day 0). Moreover, the mRNA level of DPY30 was remarkably up-regulated (Fig. 1H). the expression of DPY30 gradually increases in the continuous progression of CRC from colitis to colitis-associated CRC tumor. To sum up, DPY30 is overexpressed in CRC tissues, which has important clinical significance. And the upregulation of DPY30 might be relevant to the development of CRC.

### DPY30 knockdown suppresses proliferative capacity of CRC cells *in vitro*

**Figure 2:**
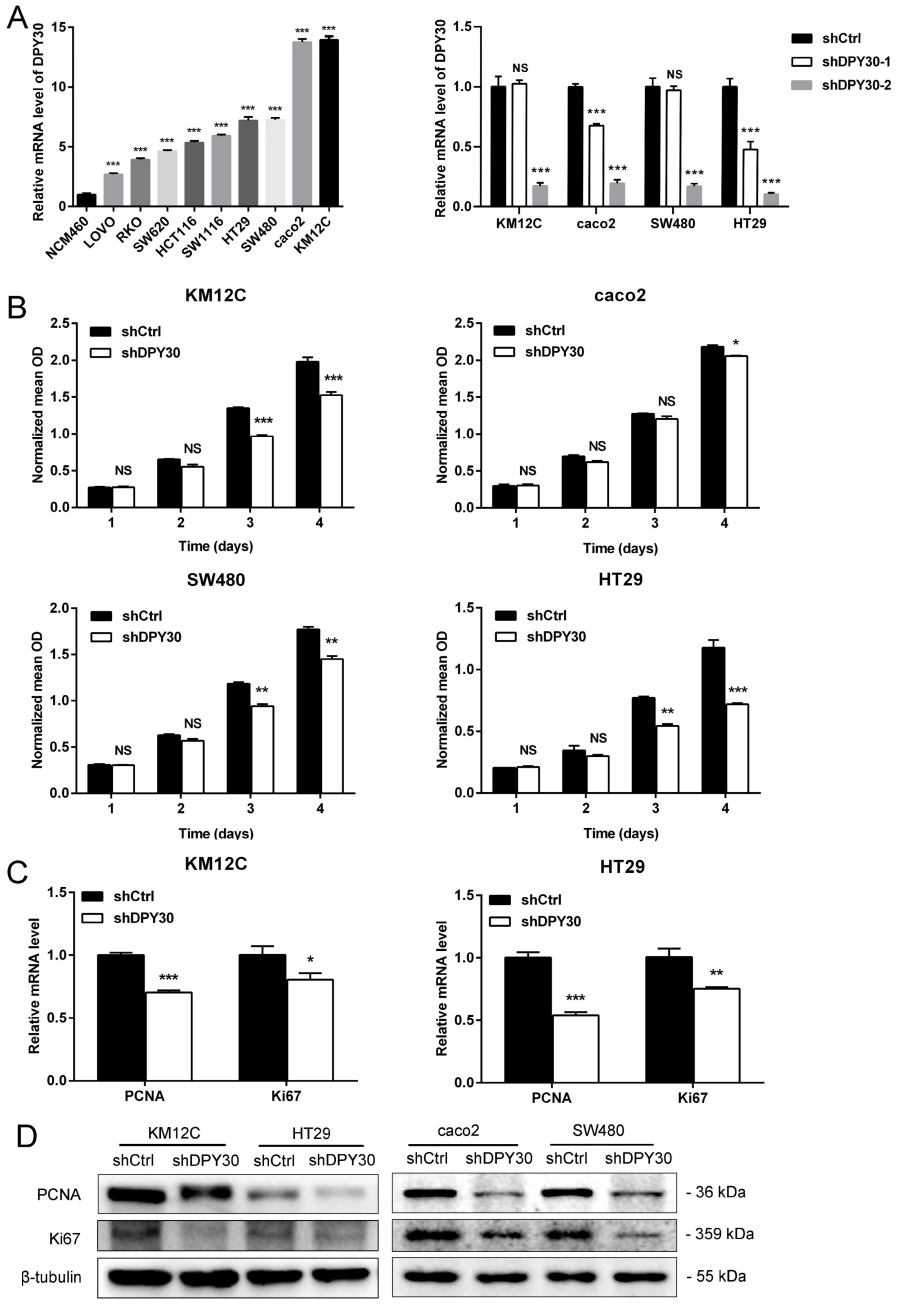
** DPY30 knockdown inhibits the proliferation of CRC cells.** (A) Left, analysis of DPY30 mRNA expression levels in a variety of CRC cell lines and normal colonic epithelial cell NCM460 by qRT-PCR. Right, efficiency of DPY30 stable knockdown in CRC cells was verified by qRT-PCR. (B) Effects of DPY30 knockdown on the proliferation of CRC cells KM12C, caco2, SW480 and HT29 measured by CCK-8 method. (C) The relative mRNA expression levels of PCNA and Ki67 in DPY30 knockdown HT29 and KM12C cells were detected. (D) The protein expression levels of PCNA and Ki67 in KM12C, HT29, caco2 and SW480 cells were determined by western blotting. Data were expressed as mean ± SEM. NS, no significant difference, *P < 0.05, **P < 0.01, ***P < 0.001.

The mRNA expression level of DPY30 was higher in 9 human CRC cell lines (LOVO, RKO, SW620, HCT116, HT29, SW480, caco2 and KM12C) than in the normal colonic epithelial cell NCM460 (Fig. 2A). to investigate the role of DPY30 in the growth of CRC cells, stable knockdown of DPY30 in HT29, SW480, caco2 and KM12C cells were constructed. Then the more effective shDPY30-2 plasmid was used (Fig. 2A, right). The proliferative capacities of HT29, SW480, caco2 and KM12C cells presented by normalized mean OD, were suppressed after DPY30 knockdown by contrast with shCtrl cells investigated by CCK8 assay, especially on 4^th^ day (Fig. 2B). And it showed that DPY30 knockdown had the most obvious and significant inhibitory effect on the growth of KM12C and HT29 cells. Therefore, we used these two cell lines as research objects for subsequent studies. Moreover, the mRNA expression level of PCNA and Ki67, two markers of cell proliferation, were decreased after DPY30 knockdown remarkably (Fig. 2C). And the PCNA and Ki67 protein expression levels in HT29, KM12C caco2 and SW480 cells were also suppressed in shDPY30 cells significantly (Fig. 2D). Therefore, these results suggested that DPY30 could be an important regulator of proliferation in CRC cells, DPY30 knockdown could suppress the proliferation of CRC cells *in vitro*.

### DPY30 knockdown induces CRC cell cycle arrest at S phase

**Figure 3:**
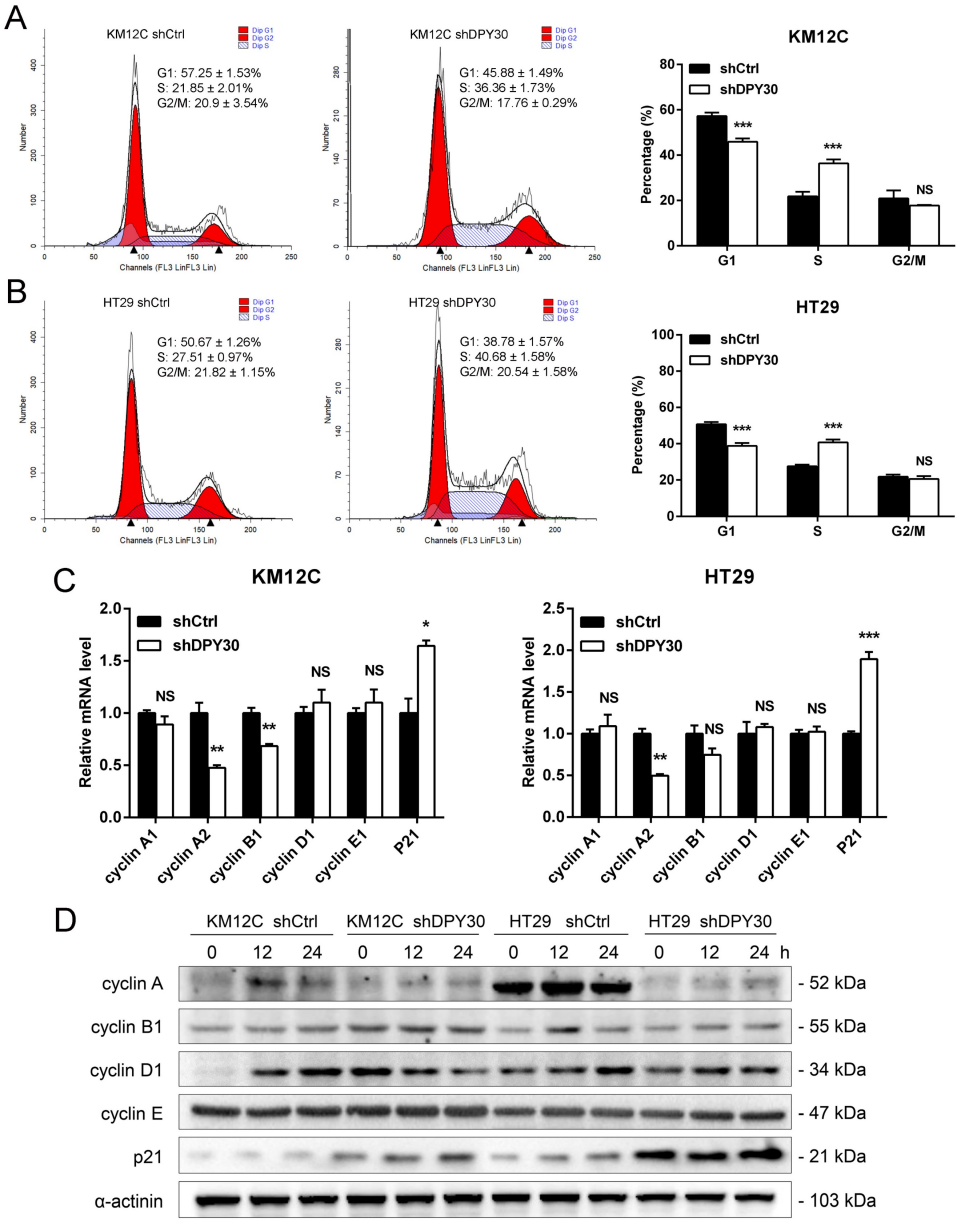
** DPY30 knockdown induces S phase cell cycle arrest in CRC cells.** (A, B) Representative cell cycle distribution of KM12C and HT29 cells examined by the flow cytometry. And the statistical histogram analysis (Right panel) for the percentage of cell cycle distribution in each phase of the cell cycle (G0/G1, S, and G2/M). (C) The relative mRNA expression levels of cyclins genes were detected (cyclin A, B1, D1, E and p21). (D) Western blotting analysis for detecting the protein expression levels of cyclins when DPY30 knockdown under the 0 h, 12 h and 24 h starvation condition. Data were expressed as mean ± SEM. NS, no significant difference, *P < 0.05, **P < 0.01, ***P < 0.001.

The rapid proliferation of tumor cells is closely related to the progression of the cell cycle, which is the typical hallmark of tumors. Flow cytometry was performed to characterize whether DPY30 was involved in the regulation of cell cycle. As shown in Fig. 3A and 3B, DPY30 knockdown significantly increased the cell population at S phase from 21.85±2.01% to 36.36±1.73% for KM12C, 27.51±0.97% to 40.68±1.58% for HT29, while the decrease of cell number at the G1 phase. Moreover, gene expression of cyclin A2 was significantly decreased and p21 was increased after DPY30 knockdown both in KM12C and HT29, whereas cyclin A1, B1, D1 and E1 were mostly impervious (Fig. 3C). Meanwhile, the cells were treated with serum starvation to synchronize the cell cycle and collected for cyclins protein detection at different time points. Obviously, the expression level of S phase-related factor cyclin A significantly decreased in HT29-shDPY30 cells and slightly decreased in KM12C-shDPY30, p21 up-regulated in both cells. whereas the protein expression level of other factors hardly changed (Fig. 3D). Taking together, these findings showed that DPY30 knockdown might induce S phase cell cycle arrest in CRC cells.

### DPY30 knockdown regulates the expression of proliferation and cell cycle related genes and biological processes based on RNA-Seq analysis

**Figure 4:**
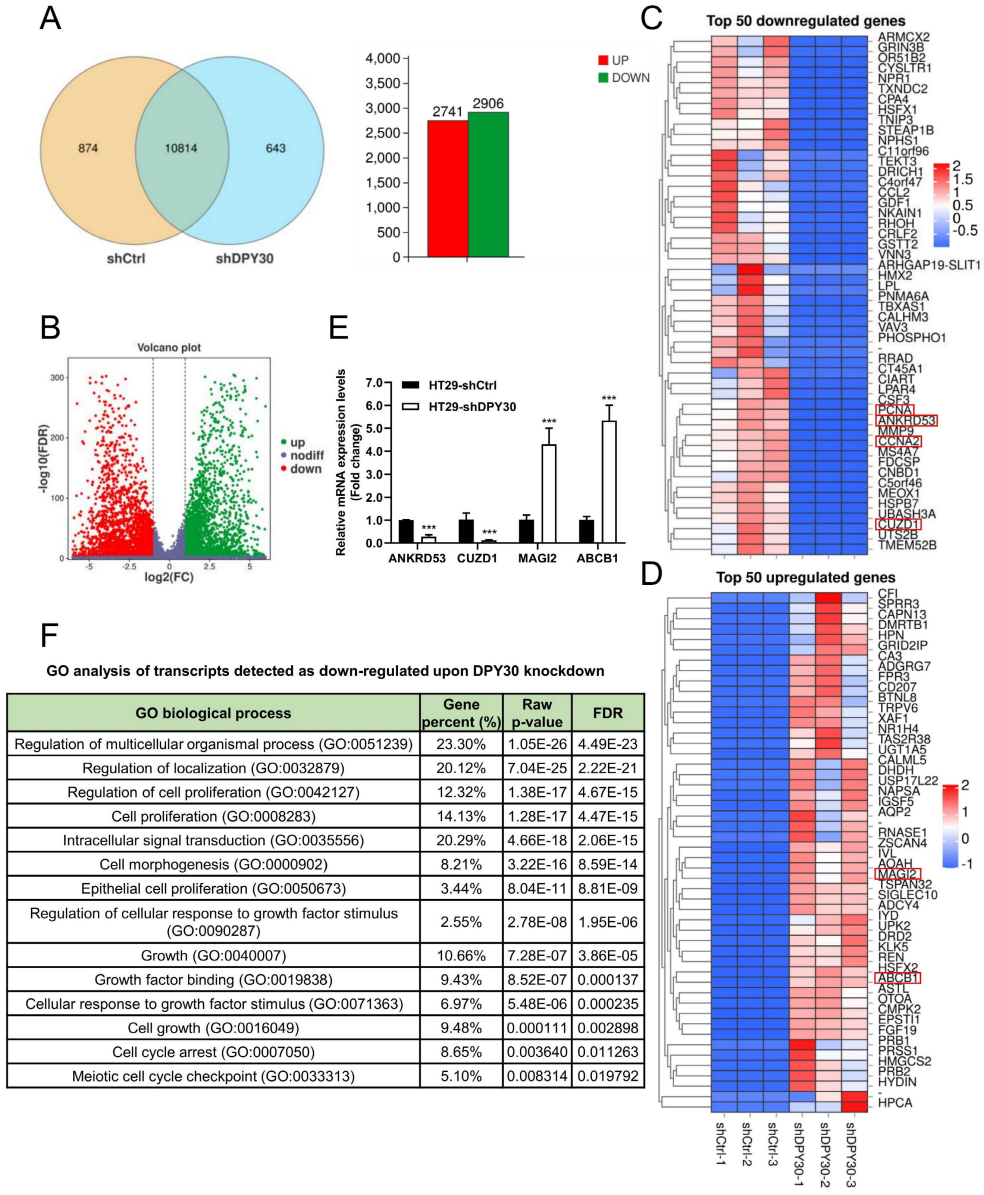
** Analysis of gene expression profile alterations upon DPY30 knockdown in HT29 cells based on RNA-Seq.** (A) Venn diagram showing the genes expressed in HT29-shCtrl and HT29-shDPY30 cells. Among these genes, 10814 are co-expressed, 2741 genes up-regulated (diagram with red bar) and 2906 genes down-regulated (diagram with green bar). The number of specifically expressed genes between the two groups is 874 and 643, respectively. (B) Volcano plot showing on the x-axis changes in gene expression levels in HT29-shCtrl and HT29-shDPY30 cells and on the y-axis the p values associated with these changes. (C, D) Heatmaps representing unsupervised hierarchical clustering of mRNA expression level depicting the top 50 downregulated (panel C) and upregulated genes (panel D) with FDR < 0.05. Each column represents the indicated sample. The genes tracked red were found and selected. (E) The relative mRNA expression levels of the other cell cycle related genes found in top 50 regulated genes (ANKRD53, CUZD1, MAGI2 and ABCB1) when DPY30 knockdown were detected by qRT-PCR. Data were expressed as mean ± SEM. ***P < 0.001. (F) Gene ontology (GO) biological process analysis for genes downregulated by DPY30's knockdown in HT29 cells (FDR < 0.05 and fold change ≥ 2).

To establish a complete quantitative and qualitative gene expression profile of CRC cells in response to altered DPY30 expression, thereby studying the potential mechanism of DPY30 in regulating cell proliferation and cell cycle progression, 3 independent transcriptomic analysis for HT29-shDPY30 and HT29-shCtrl cells were performed. We further investigated the gene expression profile of the effects of DPY30 knockdown on cell proliferation and cell cycle and the possible mechanism of DPY30. Upon setting under a corrected *P*-value (FDR) = 0.05 with fold change ≥ 2, 2741 upregulated and 2906 downregulated genes were found (Fig. 4A, B). All the differential expression genes analysis is provided in list Table S1. The top 50 downregulated genes are described in Fig. 4C and the top 50 upregulated genes are described in Fig. 4D, respectively (Table S2, S3). In particular, PCNA and cyclin A2 (CCNA2) were found in the top 50 downregulated genes (Fig. 4C). The other cell cycle regulatory genes were highlighted on heatmaps (red boxes), including ANKRD53, CUZD1, MAGI2 and ABCB. They were screened from the top 50 downregulated and upregulated genes according to the GO Process and GO Function (Table S2, S3). Then we examined the relative mRNA expression levels of ANKRD53, CUZD1, MAGI2 and ABCB1 by qRT-PCR. As shown in Fig. 4E, the expression levels of ANKRD53 and CUZD1 were significantly reduced, while MAGI2 and ABCB1 enhanced when DPY30 knockdown. The changes were consistent with RNA-Seq results and Heatmaps representation.

Then RNA-Seq analysis revealed that enriched gene ontology (GO) of cell proliferation and cell growth was significantly affected (Fig. 4F, Table S4). The GO terms of down-regulated genes were including regulation of cell proliferation (GO:0042127), cell proliferation (GO:0008283), intracellular signal transduction (GO:0035556), cell growth (GO:0016049), cell cycle arrest (GO:0007050) and so forth (Fig. 4F). This is in consistent with the results of cells growing inhibited when DPY30 downregulated (Fig. 2B, 3A, 3B). RNA-Seq data similarly confirmed that DPY30 played an important role in the regulation of cell proliferation and cell cycle, and related genes were suppressed upon DPY30 knockdown. DPY30 plays a key role in regulating cell cycle and cell proliferation presumably through regulating its potential downstream effector PCNA, Ki67 and cyclin A2 in CRC.

### DPY30 knockdown inhibits the transcriptional expression of PCNA, Ki67 and cyclin A2 via mediating H3K4 trimethylation

**Figure 5:**
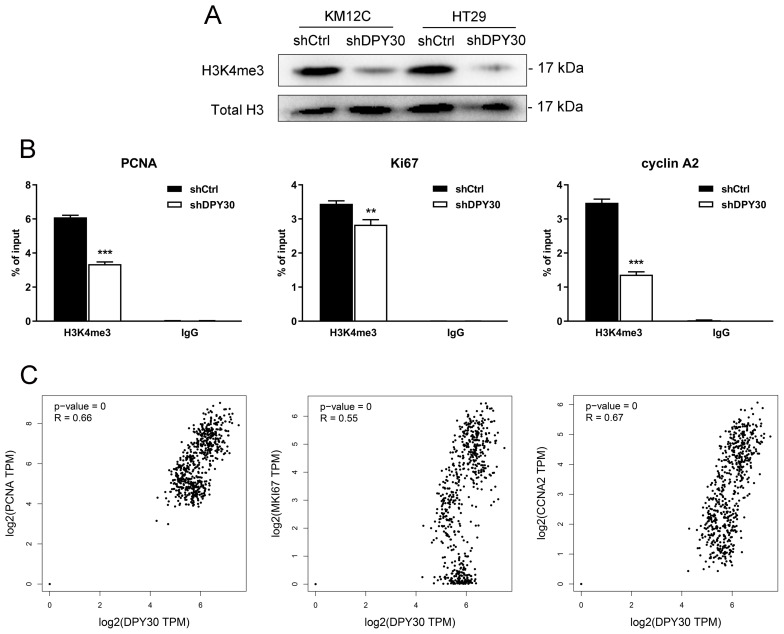
** DPY30 knockdown suppresses the establishment of H3K4me3 to PCNA, Ki67 and cyclin A2 genes then downregulates transcriptional expression.** (A) Western blotting to detected the endogenous levels of histone H3 when tri-methylated on Lys4 in KM12C and HT29 shDPY30 cells. (B) qPCR was performed to evaluate the ChIP analysis of IgG and H3K4me3 interaction status with candidate DPY30 target genes (PCNA, Ki67 and cyclin A2) after knockdown assay. H3K4me3 bindings were monitored at the promoters of PCNA, Ki67 and cyclin A2 genes. IgG antibody was included as a negative control. H3K4me3 was normalized to total H3. Values were presented as percentage of input. The results were presented as the means ± SEM of values obtained in three independent experiments. **P < 0.01, ***P < 0.001. (C) The correlations between DPY30 with PCNA, Ki67 and cyclin A2 (CCNA2) were evaluated in the GEPIA2 database. R, correlation coefficient. Positive number indicated positive correlation and negative number indicated negative correlation.

DPY30 is one of the key components of the mammalian SET1/MLL histone methyltransferase complex. Previous reports demonstrated the functions of DPY30 in regulating three different forms of H3K4 methylation, especially H3K4me3 22. Consistent with the study, obvious reductions in global H3K4me3 upon knockdown of DPY30 in KM12C and HT29 cells were found (Fig. 5A), suggesting that DPY30 might regulate gene expression by mediating H3K4me3. Then we focused on the H3K4me3 enrichment on the cell growth- and cell cycle-associated genes using ChIP assay. As determined by ChIP-qPCR shown in Fig. 5B, the establishment of H3K4me3 on promoter regions of PCNA, Ki67 and cyclin A2 were all suppressed significantly upon DPY30 knockdown. Taking this result together with Fig. 2C, 2D, 3C and 3D, it suggested that the downregulation of these genes was directly regulated by DPY30, which led to transcriptional expression and protein expression levels of genes reduced. Next, the correlations between the expression levels of DPY30 and PCNA, Ki67 and cyclin A2 in CRC were further analyzed by GEPIA 2 database (*http://gepia2.cancer-pku.cn/*, COAD and READ in TCGA expression data) (Fig. 5C), which showed that the expression of DPY30 was positively correlated with PCNA, Ki67 and cyclinA2. Taken together, these results suggested that DPY30 regulates the transcriptional expression of PCNA, Ki67 and cyclinA2 in CRC by mediating H3K4me3 level.

### DPY30 knockdown attenuates CRC tumorigenicity *in vivo*

**Figure 6:**
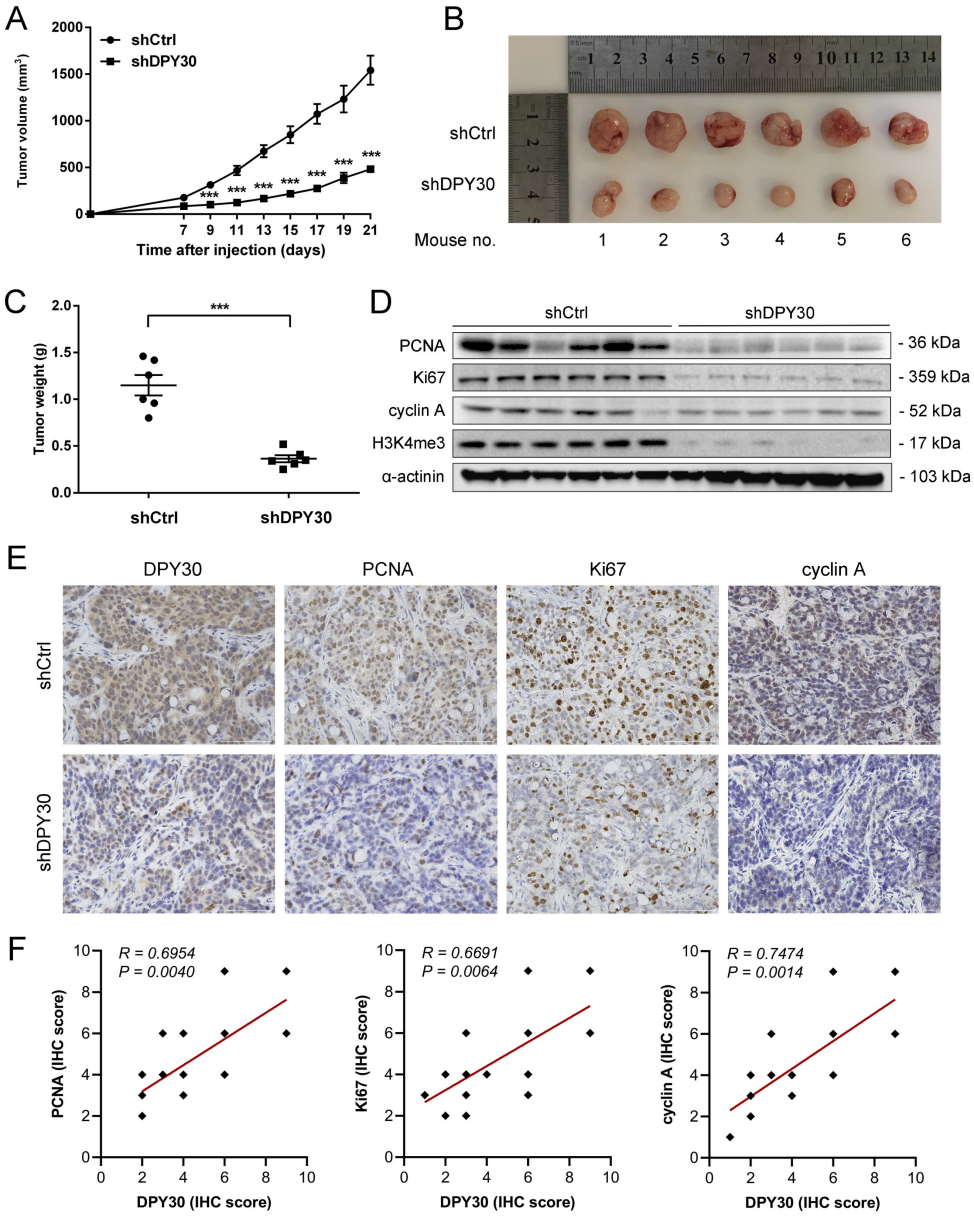
** DPY30 knockdown inhibits CRC tumor growth *in vivo*.** (A) The tumor volume growth curve of nude mice was measured from xenograft tumour models established with HT29-shDPY30 and its control cells (n = 6) every 2 days. (B) Tumors formed by subcutaneous injection photographed (C) Tumors weight were measured for statistical analysis at harvest time. (D) The changes of protein expression of PCNA, Ki67, cyclin A and H3K4me3 after DPY30 knockdown in xenograft tumor tissues by western blotting. (E) Representative IHC staining of tissue sections from shCtrl and shDPY30 tumors. Magnification, 400×. (F) The correlation between DPY30 protein expression levels with Ki67, PCNA and cyclin A protein level were measured in the animal tumor tissues respectively. The IHC score values were subjected to Pearson correlation analysis (n = 15). Data were expressed as means ± SEM of values. ***P < 0.001, between the indicated subgroups.

To further verify the effects of DPY30 on CRC tumorigenesis* in vivo*, HT29-shDPY30 cells and shCtrl cells were subcutaneously injected into BALB/c nude mice. Interestingly, the tumor volume growth of shDPY30 group was significantly suppressed in comparison with the control group from 9 days after injection (Fig. 6A). And the weight of tumor tissues was also significantly reduced (Fig. 6B, 6C). Moreover, the downregulation of DPY30 was associated with lower expression of PCNA, Ki67, cyclin A and H3K4me3 in DPY30-knockdown xenograft tumor tissues than control group appreciably (Fig. 6D, 6E). Analysis of the correlation scatter plot of the IHC score showed DPY30 protein level was positively correlated with PCNA (*R* = 0.6954, *P* = 0.0040), Ki67 (*R* = 0.6691, *P* < 0.0064) and cyclin A (*R* = 0.7474, *P* < 0.0014) in subcutaneous xenograft tumor tissues, respectively (Fig. 6F). These results indicate that DPY30 knockdown could attenuate the tumorigenicity of CRC *in vivo* through the suppression of cell proliferation and cell cycle.

## Discussion

**Figure 7:**
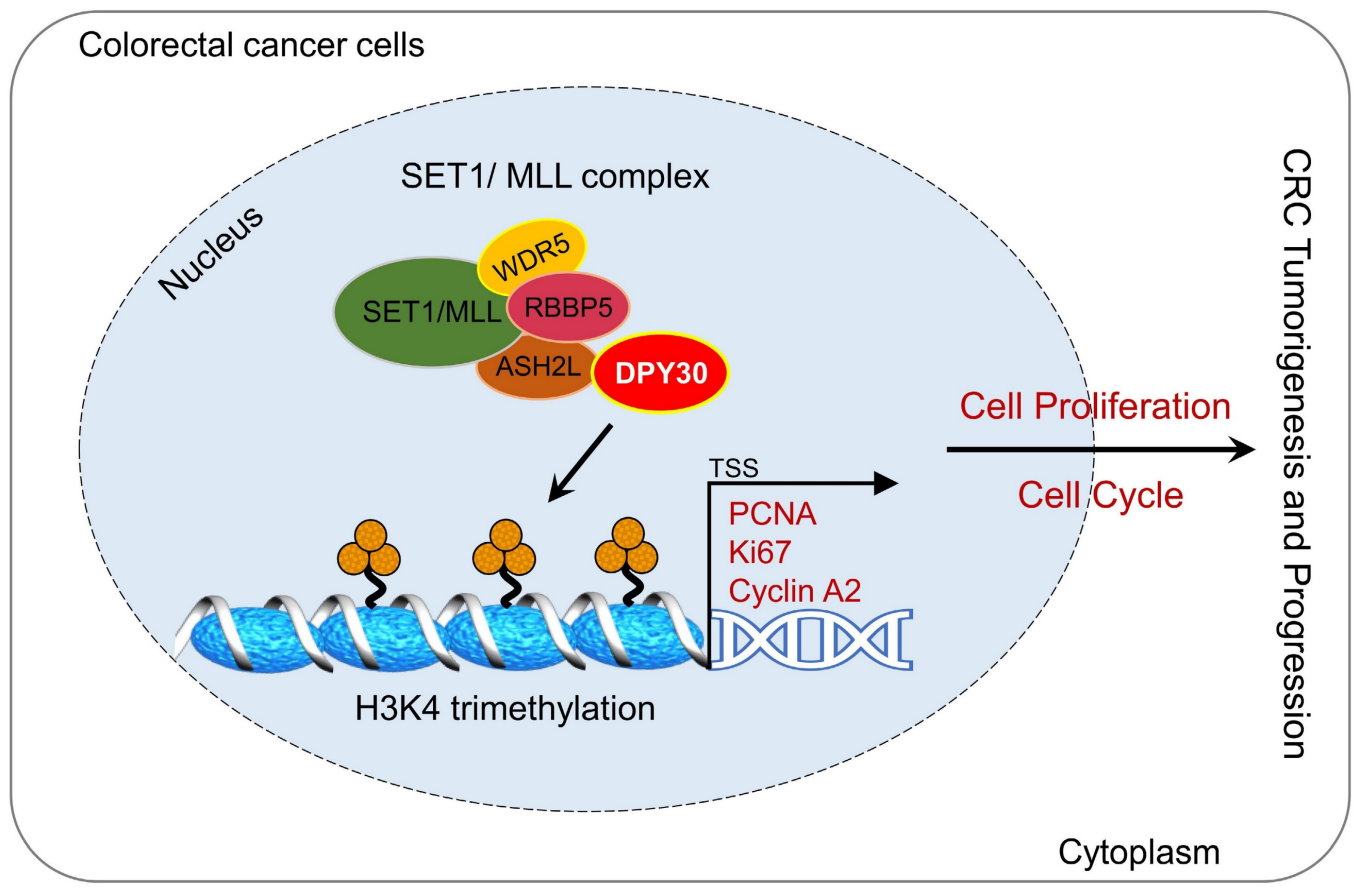
** Graphical Abstract.** Overexpression of DPY30 promotes CRC cell proliferation and cell cycle progression through facilitating the transcriptional expression of PCNA, Ki67 and cyclin A2 via mediating the establishment of H3K4 trimethylation on gene promoter regions, consequently promoting the tumorigenesis and progression of color

CRC seriously threatens health, because it is closely associated with mortality and morbidity among the general population. In this study, we found DPY30 was overexpressed in CRC cells and CRC tissues, moreover, its expression was appreciably increased with CRC development, correlated with pathological grading, tumor size, TNM stage, and tumor location. Moreover, DPY30 knockdown suppressed the malignancy of CRC cells via the proliferation inhibition and S phase arrest of the cell cycle. Given that DPY30 regulates various genes in CRC, we identified the target genes of DPY30 by RNA-Seq, which was to establish a complete gene expression profile of CRC cells in response to DPY30 knockdown. Gene ontology analysis revealed the regulatory effects on cell proliferation and cell cycle. Thereout we found the potential mechanism of DPY30 in regulating cell proliferation and cell cycle progression. Specifically, as a member of SET1/MLL complexes, the changes of DPY30 expression may affect the activity of H3K4 methyltransferases. Here, we showed a mechanistic link between DPY30 and genes related to cell proliferation and cell cycle through regulation of H3K4me3, which subsequently leads to transcriptional downregulation of PCNA, Ki67 and cyclin A2 expression. These results lead us to propose a model for DPY30 regulation of tumorigenesis in CRC (Fig. 7, Graphical Abstract).

The present study focused on DPY30, the important subunit of the human SET1/MLL complexes that was required for complete SET1/MLL methyltransferase activity. Our study found that DPY30 was highly expressed in CRC, and the cell proliferative capacity was decreased and the cell cycle was arrested when DPY30 knockdown. Therefore, we carried out the in-depth study with this breakthrough, and finally confirmed the conjecture that DPY30 regulated H3K4me3 and changes the transcription of proliferation-related genes by affecting SET1/MLL complexes via qPCR, western blot, ChIP and animal experiment. Our finding about the effect of altered DPY30 expression on CRC cells was similar to the reported function of WDR5. WDR5 depletion reduced cell viability, inhibited proliferation and H3K4me3 levels in many colon cancer cells and bladder cancer 23, 24. These data demonstrate a clear similar role for DPY30, WDR5 and other subunits in CRC. It was also mentioned in the reported that consistent with the effect of the SET1/MLL complexes in regulating cell survival, proliferation and death, subunits of these complexes were also closely related to cancer 25-27. Future studies should examine their potential to serve as a therapeutic target in cancer.

DPY30 plays an essential role in both catalyzing H3K4 trimethylation and maintaining the state of trimethylated H3K4. DPY30 regulates chromosomal H3K4me3 throughout the mammalian genome by SET1/MLL complexes 12, which indicates SET1/MLL complexes are also important to the function of DPY30. Interestingly, silencing DPY30 mainly induced the S phase cell cycle arrest. The cell cycle is regulated by cyclins and cyclin-dependent kinases 28. Cyclin A1 and cyclin A2 regulate the S to G2 phase transition 28. DPY30 knockdown obviously inhibited cyclin A2 leading to S phase cell cycle arrest. However, another study showed that knockdown of MLL1, another core component of the SET1/MLL complexes, suppressed HeLa cell proliferation by reducing the expression of cyclin B and inducing the G2/M phase cell cycle arrest 29. These data suggest that the role and mechanism of DPY30 promoting CRC cell proliferation and cell cycle are not the same as MLL1.

Moreover, the histone methyltransferase MLL1 was reported as a regulator of Wnt-driven intestinal cancer, which was highly expressed in Lgr5^+^ stem cells and CRC 30. And in our study, we found that DPY30 as its core subunit was also highly expressed in CRC. Upon the loss of MLL1, histone methylation at the stem cell promoters switches from activating H3K4 trimethylation to repression 30. DPY30 knockdown also suppressed the establishment of H3K4me3 to PCNA, Ki67 and cyclin A2 promoters based on our results. Moreover, it was found that hSETD1A and its associated H3K4me3 were upregulated in CRC. Depletion of hSETD1A inhibited CRC cells growth 31. As for its subunit, we found DPY30 knockdown inhibited the proliferation of CRC cells and induced S phase cell cycle arrest as well. The recruitment of the hSETD1A HMT complex confers promoter-associated H3K4me3 that leads to the assembly of transcription preinitiation complex and transcriptional activation 31. This suggests that there is a close positive correlation between DPY30 and SET1/MLL, and the expression level of SET1/MLL in CRC may also influence the role of DPY30 in promoting the development of CRC. Taken together, DPY30 exerts the indirect oncogenic effect through its histone methyltransferase in CRC, but SET1/MLL itself is also highly expressed in tumors. Although DPY30 and the SET1/MLL are members of the complexes, but ultimately changes in their expression have the same effect on promoter activation or repression in CRC.

For the majority of reports demonstrating the role of core subunits in development and disease, the underlying mechanism was generally considered to regulate the expression of target genes by regulating H3K4 methylation 32, 33. Previous reports showed that DPY30 directly promoted the expression of the endogenous MYC gene and was crucial for MYC-driven tumorigenesis 34. This suggests that DPY30 plays an important role in promoting the expression of oncogenes, which is consistent with the conclusion found in this study that it promotes the transcription of PCNA and Ki67, thus promoting CRC progression. Another previous study showed that DPY30 directly activated the expressions of ID proteins via H3K4 methylation 33. In this study, we found DPY30 knockdown repressed the establishment of H3K4me3 on promoters of PCNA, Ki67 and cyclin A2. As a result, the expressions of these proteins were downregulated *in vitro* and* in vivo*. The function of SET1/MLL in affecting the expression of downstream target genes is well understood 12, but previously little was known about how the complex is regulated by upstream DPY30 in CRC progression. Based on our results, the action axis of DPY30-SET1/MLL-H3K4me3-genes in CRC is concatenated, which will make the specific role of whole complexes and its subunit DPY30 in CRC more systematic and clearer.

Taken together, high expression levels of DPY30 may serve as a novel molecular marker for colorectal cancer. Besides, the correlation between DPY30 with tumor metastasis deserves to be elucidated. This question will determine whether DPY30 can be used as an independent prognostic marker and criterion for CRC patient judgment. In particular, the accumulation evidence suggested that many oncogenes and tumor suppressors influenced metabolic mode of tumors 35, 36. Many oncogenes promoted glucose consumption, thereby promoted the proliferation of cancer cells including CRC. Therefore, more studies are needed to clarify the action of DPY30-mediated tumor-promoting in CRC. In addition, future genomics/proteomics-wide studies to comprehensively explore the upstream and downstream targets or effectors of DPY30 in CRC, following to elucidate them how to affect DPY30 overexpression or whether nuclear translocation occurs, and ultimately promotes the role of DPY30 in tumorigenesis. it will facilitate basic and translational research and the development of novel therapeutic approaches, which can block colorectal cancer cells proliferation.

## Conclusions

In the present study, we highlight the important clinical significance of DPY30 and reveal that it is significantly overexpressed in CRC tissues. DPY30 knockdown inhibits cell proliferation and cell cycle progression through regulating the transcriptional expression of PCNA, Ki67 and cyclin A2 via mediating H3K4me3 on genes promoter regions in CRC (Fig. 7, Graphical Abstract). Thus, DPY30 may serve as a potential target for colorectal carcinoma.

## Supplementary Material

Supplementary table 1: shCtrl-vs-shDPY30 all annotated genes.

Supplementary table 2: Top 50 downregulated genes.

Supplementary table 3: Top 50 upregulated genes.

Supplementary table 4: Gene ontology (GO) analysis of genes downregulated upon DPY30 knockdown.

## Figures and Tables

**Table 1:** Primers designed for gene expression detection.

Application	Species	Gene	Forward primer (5'-3')	Reverse primer (5'-3')
expression	human	*DPY30*	AACGCAGGTTGCAGAAAATCCT	TCTGATCCAGGTAGGCACGAG
expression	human	*PCNA*	CCTGCTGGGATATTAGCTCCA	CAGCGGTAGGTGTCGAAGC
expression	human	*Ki67*	ACGCCTGGTTACTATCAAAAGG	CAGACCCATTTACTTGTGTTGGA
expression	human	*cyclin A1*	GAGGTCCCGATGCTTGTCAG	GTTAGCAGCCCTAGCACTGTC
expression	human	*cyclin A2*	CTTCACCAGACCTACCTCAAAG	GGTGGGTTGAGGAGAGAAAC
expression	human	*cyclin B1*	AATAAGGCGAAGATCAACATGGC	TTTGTTACCAATGTCCCCAAGAG
expression	human	*cyclin D1*	GCTGCGAAGTGGAAACCATC	CCTCCTTCTGCACACATTTGAA
expression	human	*cyclin E1*	AAGGAGCGGGACACCATGA	ACGGTCACGTTTGCCTTCC
expression	human	*P21*	TGTCCGTCAGAACCCATGC	AAAGTCGAAGTTCCATCGCTC
expression	human	*ANKRD53*	GGAGCACAACTACCTGATTGA	CTTGGTATTGGAGACCAGAGAG
expression	human	*CUZD1*	CATCTCCAACCTACGACCTAATC	GGAATCTCCCATAGTGTCCAAA
expression	human	*MAGI2*	GACAGCGGGTGAAACAAATAC	TGGCTCAGGTTCTGTACATTC
expression	human	*ABCB1*	TGCTGGTTGCTGCTTACA	GCCTATCTCCTGTCGCATTATAG
expression	human	*α-tubulin*	CCAAGCTGGAGTTCTCTA	CAATCAGAGTGCTCCAGG
expression	human	*β-actin*	CATGTACGTTGCTATCCAGGC	CTCCTTAATGTCACGCACGAT
expression	mouse	*DPY30*	AGTACGGGCTCACAGACA	GATAAGATGCTAGGAACTCGATGG
expression	mouse	*β-actin*	CACTGTCGAGTCGCGTCCA	TGACCCATTCCCACCATCAC
shCtrl	human	scramble	CCGGGGCTACGTCCAGGAGCGCACCCTCGAGGGTGCGCTCCTGGACGTAGCCTTTTTG	AATTCAAAAAGGCTACGTCCAGGAGCGCACCCTCGAGGGTGCGCTCCTGGACGTAGCC
shDPY30-1	human	*DPY30*	CCGGGACCACCAAATCCCATTGAATCTCGAGATTCAATGGGATTTGGTGGTCTTTTTG	AATTCAAAAAGACCACCAAATCCCATTGAATCTCGAGATTCAATGGGATTTGGTGGTC
shDPY30-2	human	*DPY30*	CCGGCACAGTTTGAAGATCGAAACCTCGAGGTTTCGATCTTCAAACTGTGTTTTTG	AATTCAAAAACACAGTTTGAAGATCGAAACCTCGAGGTTTCGATCTTCAAACTGTG
ChIP	human	*PCNA*	AGAAAGTTTCCAGCCACGAA	CGGGGGAATGTTAAGAGGAT
ChIP	human	*Ki67*	CTCTTGGGAGCATGAGAATGAG	GGTGAAGTGAATGGAGCCTAAA
ChIP	human	*cyclin A2*	ACTAGACGTCCCAGAGCTAAA	TGTCCGAAGGCTGACTCTAA

**Table 2:** Expression of DPY30 protein in different colorectal tissues.

Tissue type	n	DPY30 stain grades				*X^2^*	*P*
		0	1	2	3		
Tumor	94	6	33	48	7	58.773	< 0.0001*
Paratumor	86	34	45	7	0		

^*^Indicates statistical significance.

**Table 3:** Associations between DPY30 expression and clinicopathological characteristics of CRC patients.

Characteristics	Variable	N	DPY30		*X^2^*	*P*
			Low	High		
Age						
	< 60	28	11	17	0.079771	0.7776
	≥ 60	66	28	38		
Gender						
	Male	48	19	29	0.14681	0.7016
	Female	46	20	26		
Pathological grading						
	Ⅰ	3	0	3	125	0.0348*
	Ⅰ-Ⅱ	16	6	10		
	II	60	22	38		
	II-III	9	5	4		
	III	5	5	0		
	Ⅲ-Ⅳ	1	1	0		
Tumor size						
	d ≤ 4	28	12	16	7.9052	0.0192*
	4 < d ≤ 6	40	19	21		
	d > 6	20	2	17		
T category						
	T1	1	0	1	2.053	0.5622
	T2	10	4	6		
	T3	51	19	32		
	T4	32	16	16		
N stage						
	N0	60	24	36	0.15212	0.9268
	N1	25	11	14		
	N2	9	4	5		
M stage						
	M0	89	37	52	0.0048251	0.9446
	M1	5	2	3		
TNM stage (AJCC)						
	Stage I	10	7	3	8.5173	0.0365*
	Stage II	48	19	29		
	Stage III	31	13	16		
	Stage IV	5	0	7		
Tumor location						
	Ascending colon	34	18	16	13.525	0.019*
	Colon hepatic flexure	6	4	2		
	Transverse colon	5	4	1		
	Colon splenic flexure	3	1	2		
	Descending colon	8	2	6		
	Sigmoidal and rectum	36	8	29		
The pathologic types						
	Tubular adenocarcinoma	7	2	5	2.6843	0.4429
	Adenocarcinoma	83	34	49		
	Mucous adenocarcinoma	3	2	1		
	Signet ring cell carcinoma	1	1	0		
Positive lymph nodes						
	n ≤ 3	84	35	49	2.8353	0.4178
	3< n ≤ 7	7	4	3		
	n > 7	2	0	2		

^*^Indicates statistical significance.

## Abbreviations

CRC: colorectal carcinoma
H3K4me1: Mono-Methylation of lysine 4 on histone H3 protein subunit
H3K4me2: Di-Methylation of lysine 4 on histone H3 protein subunit
H3K4me3: Tri-Methylation of lysine 4 on histone H3 protein subunit
shDPY30: DPY30 knockdown
CCK-8: cell counting kit-8
IHC: Immunohistochemistry
RT-qPCR: Quantitative Real-time Polymerase Chain Reaction
PBS: phosphate buffer saline
ChIP: Chromatin immunoprecipitation
RNA-Seq: RNA Sequencing
GO: Gene Ontology
GEPIA: Gene Expression Profiling Interactive Analysis
AOM/DSS: Azoxymethane/ Dextran Sulfate Sodium
ROC: receiver operating characteristic
AUC: areas under the curves
CCNA2: Cyclin A2
PCNA: Proliferating cell nuclear antigen
